# Free lipoproteins from *Bifidobacterium longum* alleviate osteoarthritis through modulation of the gut microbiome

**DOI:** 10.20517/mrr.2023.12

**Published:** 2023-05-11

**Authors:** Famara Sane, Frank Piva, Marie-Bénédicte Romond

**Affiliations:** ^1^ULR3610, Centre Hospitalier Universitaire de Lille, Lille 59000, France.; ^2^ULR3610, Université de Lille, Lille 59000, France.

**Keywords:** Lipoproteins, bifidobacteria, osteoarthritis, gut bacteria

## Abstract

**Aim:** The “gut-joint” axis is suspected to be involved in the pathophysiology of osteoarthritis (OA). The present study aims at investigating the potential of lipoproteins (Lpps) secreted by *Bifidobacterium longum* to alleviate OA progression in the rat.

**Methods:** Experimental OA was induced in rats harbouring Schaedler Flora maintained in SPF conditions. Two weeks post-injection, 20 rats were randomized to water (*n* = 10) or 0.3 mg/L Lpps solution (*n* = 10). Weight and food intake were monitored for 6 weeks. At sacrifice, joints were scored using macroscopic and histological criteria. Serum LPS, Schaedler flora as well as selected intestinal bacteria were analyzed.

**Results:** Lpps intake prevents OA progression. The protected rats showed a significant increase in lactobacilli along the intestine as well as in *Mucispirillum schaedleri* in the colon and a significant decrease in *Parabacteroides goldsteini* and *Akkermansia* in caecum and colon, respectively. There was no significant difference in serum lipopolysaccharide or bacteria translocating in Peyer's patches. Labelled Lpps were not detected in bone marrow of the OA joint. The principal component analysis points out that OA prevention is primarily associated with bacteria involved in the tryptophane degradation pathway and SCFA formation.

**Conclusion:** In rats deprived of bifidobacteria, intake of *B.longum* Lpps prevented OA development and modulated the intestinal microbiome with a possible impact on the bacterial end-products. The link between Lpps and the gut microbial metabolome warrants further investigation.

## INTRODUCTION

Bacterial lipoproteins are secreted membrane-anchored proteins characterized by a lipobox motif^[1]^. This lipobox motif directs post-translational modifications at the conserved cysteine through the consecutive action of three enzymes: diacylglyceryltransferase (Lgt), pro-lipoprotein signal peptidase (LspA) and N-acyltransferase (Lnt), which results in di- or triacylated forms. Lipoproteins (Lpps) are abundant in all bacteria including bifidobacteria, but only a few are released from the cell wall during bacterial growth^[2,3]^. Once released, they exhibit a range of biological activities, from immunoregulatory to anti-viral properties^[4,5]^. *In vivo*, intake of bifidobacterial lipoproteins-containing extracts induces changes in the microbiota balance with the promotion of the gut bifidobacteria and a decrease in *Bacteroides* and clostridia^[6-8]^. In addition to the gut microbiome modulation, administration of the aggregated lipoprotein forms to mice harboring the microbiome from an arthritis donor abrogate the altered antigen presentation genes expression in splenic CD11c+ cells^[3]^. The results emphasized the inflammatory potential of the arthritis microbiome towards the otherwise healthy host and the beneficial effect of Lpps to restore balance. Above all, it opens up new avenues for possible protection against microbiome-related osteoarthritis symptoms.

The present study aims at defining whether Lpps can prevent osteoarthritis (OA) progression. OA is the most common form of arthritis and a major cause of disability worldwide. OA affects the whole joint, leading to cartilage degradation, synovial inflammation and subchondral bone remodelling^[9]^. Indeed, a link between dysbiosis and OA progression is observed in the context of obesity. The data in animal models indicate that cartilage damage and degeneration in diarthrodial joints are related to microbial shifts in the gut that can be alleviated by the administration of prebiotics or probiotics^[10-12]^. Moreover, the germ-free status partially protects mice from OA^[13]^.

To address the question of gut-joint axis and the possible protection by Lpps, we induced OA in Altered Schaedler Flora (ASF) associated rats kept in specific pathogen-free (SPF) conditions. Eight weeks after injection of monoiodoacetate (MIA), the intestinal colonization and translocation to Peyer’s patches of selected bacteria was sought by using qPCR quantification in Lpps-treated and untreated rats. An untreated sub-group of MIA and sham rats were given labelled Lpps prior to sacrifice and antigen-presenting cells (APC) were collected from bone marrow and spleen. Our results provide evidence that Lpps intake conveys protection against OA development and induces shifts to gut bacterial colonization.

## MATERIAL AND METHODS

### Preparation and characterization of Lpps

*Bifidobacterium longum* subsp *longum* CBi0703 strain (Bl CBi0703) was grown in an anaerobic chamber for 48 h hours at 37 °C in a pre-reduced broth (2 L) containing lactose (70 g/L) and partially digested whey proteins as previously described^[3]^. Bifidobacteria were enumerated at 0 h (7.4 log cfu/mL), 24 h (8.9 log cfu/mL) and 48 h (8.3 log cfu/mL)^[6]^. Monitoring of broth acidification showed a drastic pH drop to 4.5 within 24 h. The supernatant was collected by centrifugation (11.000 x g for 20 min) and filtrated using a 10 kDa cut-off membrane (Millipore) to remove acetic and lactic acids. The 10 kDa retentate was then filtrated using a 100 kDa cut-off membrane to remove the broth proteins. The resulting retentate was collected and diafiltrated extensively against sterile water. The residual broth proteins were looked for by subjecting the lyophilized retentate to gel filtration chromatography on a Superdex200® column (Sigma-Aldrich, St Quentin Fallavier, France)^[3]^. The bifidobacterial Lpps peak corresponded to 90% of the lyophilized powder.

Medium Aggregate Extract (MAE) used as a control was produced as follows: the unfermented broth was acidified at pH 4.8 by adding a mix of 2M Lactic/ 3M Acetic acid, then incubated 48 h at 37 °C in anaerobic conditions. The acidified broth supernatant was collected by high-speed centrifugation, and then concentrated as described above.

### OA induction

Experiments were carried out in accordance with the EU directive 2010/63/EU and approved by the Ethical Committee N°120 (n° 4160-201602171657248). Male Crl:CD (SD) rats were purchased from Charles River (Saint Germain Nuelles, France). The colony of rats was originally started from foundation colony rats that had only eight species of bacteria, the Charles River Altered Schaedler Flora and later bred in SPF housing facility^[14]^. The 8 species are: *Clostridium* sp (ASF 356), *Lactobacillus intestinalis* (ASF360), *Ligilactobacillus murinus* (ASF 361), *Mucispirillum schaedlerii* (ASF 457), *Eubacterium plexicaudatum* (ASF492), *Pseudoflavonifractor* sp (ASF 500), *Schaedlerella arabinosiphila* (ASF502), *Parabacteroides goldsteinii* (ASF519).

Experimental OA was induced in rats weighing 125-150g with a single intra-articular injection of MIA (3 mg/50 uL, Day 0) into the right hind knee joint. The sham group received 50 μL of sterile PBS into the right hind knee joint. Lameness recovery occurred within 7-8 days. Rats were then transferred to the SPF housing facility. They were acclimated to the new environment seven days before the study’s commencement. They were housed in type IV cages (2/cage), with ambient temperature 20-25 °C, a 12:12 light/dark cycle (lights on at 0800 h) and access to food (sterile RO3-40 diet-Safe-Diets, Augy, France) and sterile water during the dark period.

To monitor Lpps or water intake, rats were isolated at 1800 h and placed individually into a new experimental Plexiglas cage with a background shelf. Bottles with a certain volume of 0.3 mg/L or water were placed each evening in the feeders, the remaining volumes being recorded the next morning. The treated group received the Lpps solution 5 nights per week for 6 weeks. The procedure was applied from D15 after surgery until the end of the study.

Rats were weighed daily. Energy intake was estimated daily by weighing the pellets left in the feeders each morning. Animals were euthanized at the end of the study (8-9 weeks post-surgery), with intraperitoneal injections of sodium pentobarbital (CEVA santé animal, Libourne, France).

### Macroscopic and histopathological scoring

Whole knee joints from both hind paws of rats were dissected. Cutaneous and subcutaneous tissues were removed to expose the musculotendinous structure and patella for macroscopic staging. Macroscopic scoring was performed as follows: 0 no damage (no morphological differences as compared with the left untreated paw), 1 moderate damage, 2 severe damages (obvious joint degeneration and inflammation characterized by hyperplasia of synovial membrane and swelling of articular joint). Specimens were fixed immediately in 7.5 % formaldehyde and then decalcified by immersion in Osteomoll® solution (Merck, Molsheim, France) for 7 days. After dehydration by a series of ethanol immersions, samples were embedded in paraffin and then frontally sectioned. Glass slides were mounted with 10 µm tissue sections. Deparaffinization was made by immersions in xylene and ethanol. The slides were subjected to hematoxylin and eosin staining. For each block, a minimum of five slides was obtained to minimise sampling error. Each slide was examined under a light microscope. Histological scoring was carried out by two blinded and independent observers from the following criteria: the thickness of articular cartilage, continuity of surface cartilage, and infiltration of mononuclear cells.

### Bacterial enumeration in organs

Organs (distal ileum, caecum and colon fractions, spleen, Peyer’s patches) suspended in 9 mL pre-reduced Ringer solution (Solabia, Pantin, France) supplemented with cysteine HCl (0.03 %) (VWR, Fontenay-sous-Bois, France) were weighted and kept frozen until total DNA extraction. After thawing, the total DNA was extracted using the Nucleospin Tissue kit (Macherey Nagel, Hoerdt, France). DNA content was determined at 260-nm wavelength using Biophotometer Plus (Eppendorf, Montesson, France). ASF bacteria and selected bacteria possibly involved in OA development^[12,15]^ were enumerated by qPCR^[16]^ as described in the supplementary material [Supplementary Table 1]. Plasma lipopolysaccharide was measured using Charles River Endosafe kit following the manufacturer’s instructions.

### Isolation of Antigen-Presenting Cells (APC)

Femurs and tibias were flushed into 20 mL 50 mM PBS. Spleen fraction was dilacerated into 50 mM PBS. Cell suspensions were pass-through a 70 µm sieve and washed 3 times with a solution of 50 mM PBS, 2 mM EDTA, and 2 % BSA. Antigens presenting cells (APC) were isolated from total cells using isolation columns with Anti-MHC Class II (OX6) MicroBeads (Miltenyi Biotec, Paris, France). Both APC(+) and APC(-) were counted using Malassez cells following Trypan blue staining to exclude dead cells.

### Uptake of labelled Lpps in sham and OA induced rats.

Tetramethylrodamine (TRITC)-labelled Lps and medium extract (MAE, see supplementary methods for labelling) were administrated to a subgroup of sham and MIA-induced rats by adding at the end of the survey 1.5 or 15.0 mg/L of Lpps or MAE with 10 µL of the cell-permeable fluorescent dye 5’-carboxyfluorescein succinimidyl ester (CFSE) in the bottles, 12 h before sacrifice. The remaining volumes were recorded in the morning and the animals were sacrificed within 30 min. Spleen and BM cells were collected and APCs were isolated as described above. Twenty µL cell suspensions were fixed with 4 % PFA and Fluor Save ™ (Merck Millipore) on glass slide. Labeled cells were viewed on an Eclipse E600 (Nikon) confocal microscope.

### Statistical analysis

PCAmix, combining a principal component analysis (PCA) with a multiple correspondence analysis (MCA), was performed using qualitative (diet, OA) and quantitative (bacteria located in the various organs, APC counts, weight) variables (XLstat, 2019). Partial least squares regression model (PLS-R) was used for multivariate analyses with bacteria and APC as the dependent variables. The data were visualized using a 99% confidence ellipse. ANOVA, ANCOVA and Kruskal-Wallis with post-hoc tests were used for multiple group univariate analyses, along with chi-squared (χ^2^) and Fisher tests as indicated in the text. Bivariate correlations were determined using Spearman’s Rho.

## RESULTS

### Lpps administration prevents OA development

**Figure 1:**
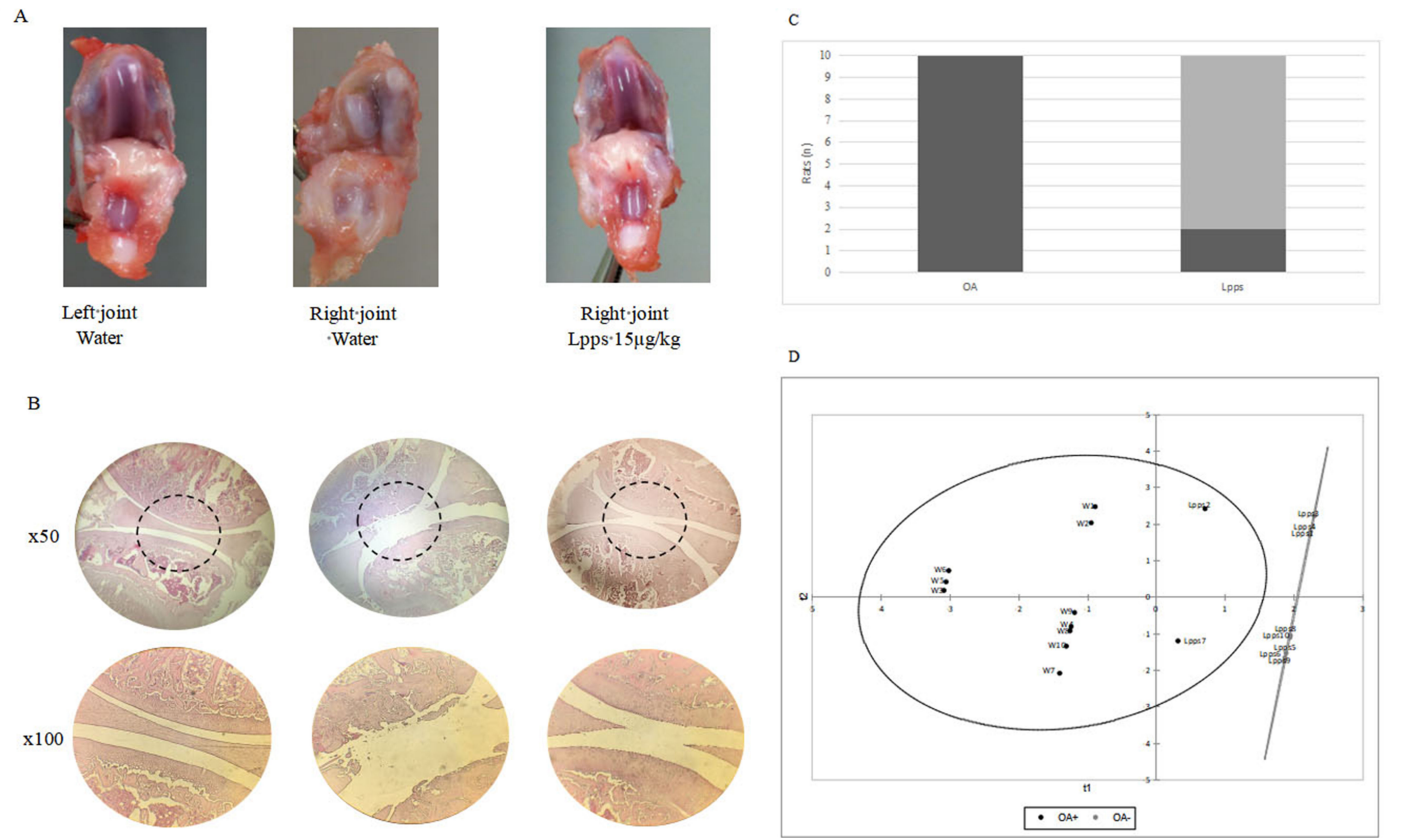
Development of OA according to Lpps administration. Representative images of macroscopic degradation. (A) and of H&E- stained joints; (B) of left knee (no injection), right MIA-injected joint from untreated and Lpps treated rat; (C) significant lower prevalence of OA in Lpps treated group (Fisher’s exact test; *P* < 0.008); (D) rat discrimination by bacteria profiling according to OA using PSL-R analysis. BC: treated rats; W: water-drinking rats; dark circle: OA rats; grey circle: healthy rats.

Osteoarthritis (OA), as defined by macroscopic and histological scoring [Figure 1A and 1B], did not develop in 8 rats out of 10 receiving 0.3 mg/L (16.4 ± 3.3 µg/kg) 5 days per week for 6 weeks. In contrast, all rats belonging to the water drinking group developed OA [Figure 1C].

### Rats responsive to Lpps treatment are clustered apart from the OA rats

PLS-R analysis of weight, APC numbers and bacterial counts data shows clustering by rat group according to OA development [Figure 1D]. Plots corresponding to the two unresponsive Lpps rats are localized between the water drinking group prone to OA development and the responsive Lpps group suggesting that an optimal balance between the selected variables is associated with the protection.

**Figure 2:**
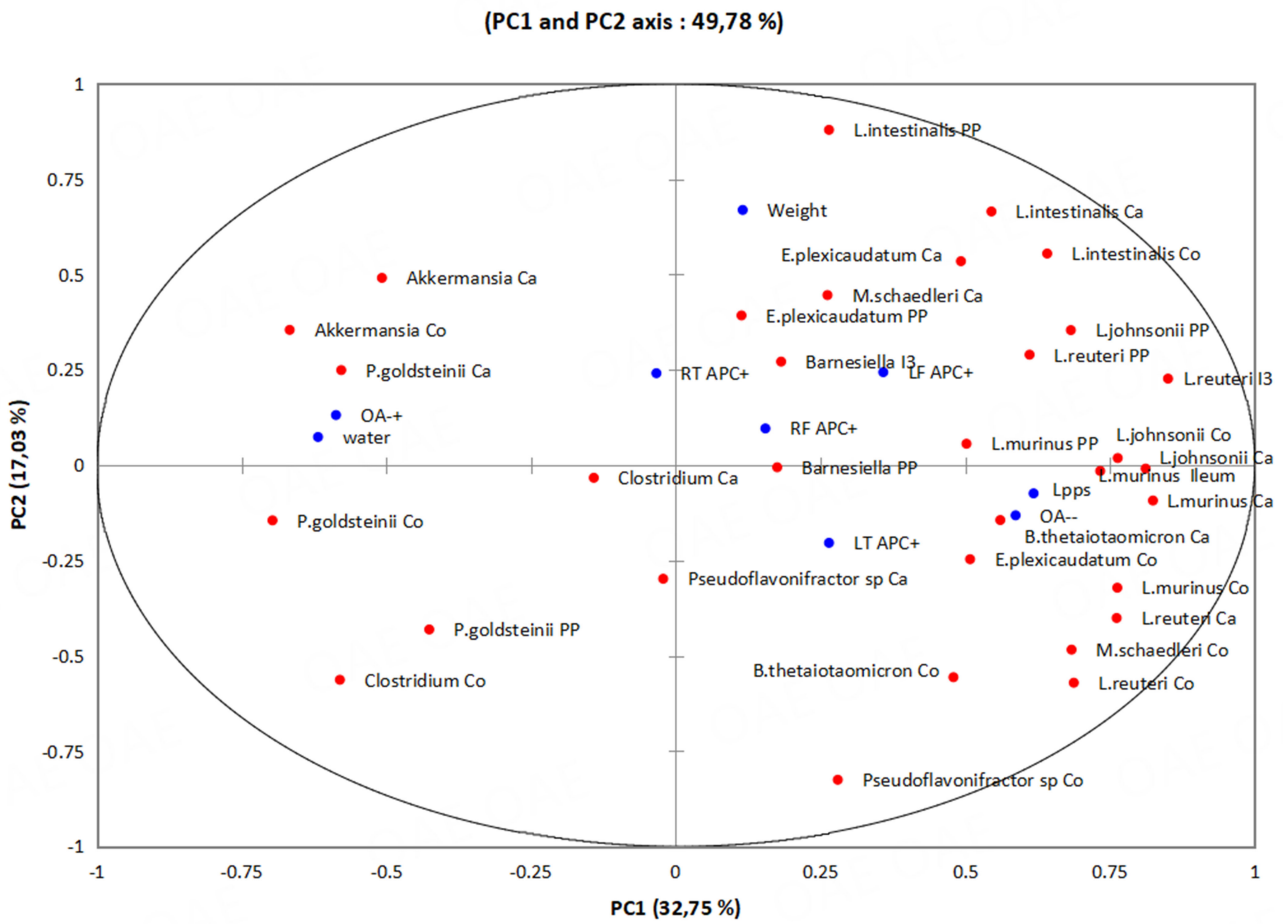
PCA illustrating the contribution of bacteria and antigen-presenting cells to OA progression according to the Lpps treatment. The dataset comprises bacterial counts in the digest tract and Peyer’s patches, body weight and bone marrow antigen-presenting cells (red circles) from 10 untreated and 10 Lpps treated rats. Blue circles correspond to the qualitative variables (i.e., OA progression and Lpps/water intake).

Principal component analysis of the data from the whole group draws attention to two bacteria strongly associated with the protective effect, i.e., *Bacteroides thetaiotaomicron* and the butyrate-producing *Eubacterium plexicaudatum* [Figure 2]. *Ligilactobacillus murinus* in the distal ileum contributed to the Lpps effect as well as *Lactobacillus johnsonii* localized in the caecum and colon. Additionally, *Parabacteroides goldsteinii* colonizing the lower part of the digestive tract is associated with OA progression.

Moreover, PCA of data excluding the two unresponsive Lpps-treated rats linked as well *B. thetaoiotaomicron*, *E. plexicaudatum* and *P. goldsteinii* to the regulation of OA onset. OA progression was not related to weight. The number of APC (+) in bone marrow does not either contribute to OA onset.

### A few bacteria are targeted by Lpps intake

To further investigate Lpps effect on the selected bacteria, counts were compared according to the diet and OA development using ANOVA and Bonferroni post-hoc test [Table 1 and Supplementary Table 2]. In the whole group (i.e., 10 control rats and 10 Lpps-treated rats), *L. murinus* and enterococci showed significant expansion in the distal ileum of the Lpps-protected rats. On the contrary, rats developing OA exhibited higher counts of *Akkermansia sp*. in the colon.

**Table 1:** Bacteria affected by Lpps and/or OA development

**Rats** **Organ**	**Bacteria**	**Whole group**		**Lpps responsive/water control rats**	
		**promoting effect**	P^*^	**promoting effect**	***P***
**Distal ileum**	*Ligilactobacillus murinus*	H^**^	0.034	H/Lpps	0.048
	enterococci	H	0.025	-	NS
	*Limosilactobacillus reuteri*	-	NS	H/Lpps	0.033
**Caecum**	*Lactobacillus johnsonii*	-	NS	H/Lpps	0.02
	*Parabacteroides goldsteinii*	-	NS	OA/Water	0.021
**Colon**	*Akkermansia sp.*	OA	0.047	OA/Water	0.01
	*L.johnsonii*	-	NS	H/Lpps	0.008
	*L.reuteri*	-	NS	H/Lpps	0.023
	*Mucispirillum schaedleri*	-	NS	H/Lpps	0.046

^*^P value corresponding to intergroup comparison (ANOVA, Bonferonni post-hoc test) are given for the whole group (Total *N* = 20, 10 Lpps treated, 10 water drinking rats) and for the responsive group (R *N* = 18, 8 Lpps treated, 10 water drinking rats); ^**^promoting conditions are listed as H (healthy), OA (OA development), Lpps (Lpps treatment), Water (untreated animals).

The exclusion of the two unresponsive rats unveiled more shifts. Healthy status was still associated with higher *L. murinus* counts in the distal ileum, together with *Limosilactobacillus reuteri*. The rats responsive to Lpps intake harbored more *L. johnsonii* and less *P. goldsteinii* in the caecum than the untreated rats. *Mucispirillum schaedleri* was also promoted in the colon of protected rats, whereas *Akkermansia sp*. colonization was reduced. Additionally, no difference was seen in plasma LPS.

### Uptake of Lpps by BM cells

The possible uptake of broth milk proteins retained during Lpps extraction (less than 10 % contamination) was checked by administrating TRITC-labeled milk aggregate extract (MAE) as a control to OA rats. In addition, two dosings were used for tracing the lipoprotein core, with only a few amino acid sequences being accessible for labeling. The highest doses of TRITC-labeled Lpps ingested by sham or OA water-drinking rats ranged between 0.83-1.13 mg/kg. The rats receiving 1.5 mg·Lpps/L were dosed with around 140 µg/kg, which is still 7 times higher than the mean dose received by Lpps-treated rats over the survey (21 µg/kg).

No signal of Lpps or MAE was detected in spleen cells. No signal of Lpps was neither observed in APC (+) from OA rats receiving the lowest dose, which contrasted with the detection of a red signal in BM from the right tibia of MAE-treated OA rat.

**Figure 3:**
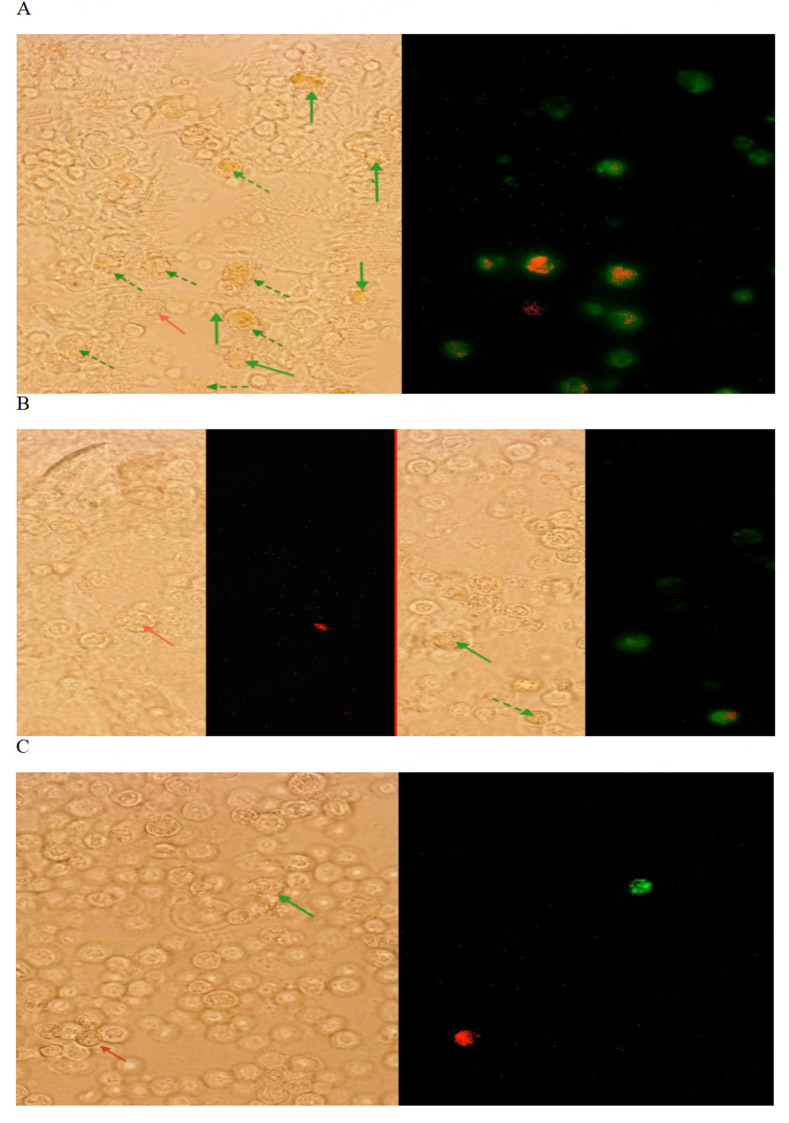
Translocation of bacteria (green) and Lpps (red) to bone marrow cells from MIA-induced (A, B) or sham (C) rats. The two fluorescent readings (red: TRITC-labeled Lpps or MAE; green: CFSE labeled bacteria) were merged to detect cells capturing Lpps or MAE (red arrow), bacteria (green arrow) or both bacteria and Lpps (dotted green arrow). TRITC-Labelled Lpps were given at the dose ranging 0.8-1.1 mg/Kg body weight (A, C). Uptake of the single labelled Lpps was detected (red arrow) in APC+ from the left femur of OA rat (A) or in APC(-) compartment from the right tibia of sham rat (C). TRITC-labelled MAE was detected either in APC (+) or APC (-) compartment of femur or tibia (B: APC(-) compartment from the right tibia). Lpps was not detected following administration of the low dose (140 µg/kg) although bacteria were detected in BM APC(+) from right joint (3.5%-6.6 % total APC(+) and the left one [8.8%-12.4 % total APC (+)].

Intake of the highest Lpps dose allows for the detection of the sole red signal in 0.3 % APC(+) cells in the left femur from a single OA rat [Figure 3A]. Most of the APCs exhibited red and green signals that indicate uptake of intestinal bacteria loaded with Lpps.

TRITC-labeled MAE administrated at a high dose led to the detection of the red signal in APC(+) cells in BM from the left (0.2 %) and right (0.3 %) femurs [Figure 3B].

In sham animals, spleen cells were devoid of a single red signal. Cells harboring red fluorescence alone were detected in BM APC(-) cells from at least three bone samples (right tibia and femur, and left tibia or femur), suggesting that BCs uptake at high dosing occurs more readily in healthy animals, likely through a different route [Figure 3C].

## DISCUSSION

The aim of this study was to explore the potential of bifidobacterial Lpps to prevent OA progression. There are obvious limitations with animal models, only mimicking parts or stages of the disease, with no model completely reproducing human OA complexity. Moreover, the reduction in the microbiome richness oversimplifies the gut-joint axis. Still, rats developed OA following MIA injection. MIA, an inhibitor of glyceraldehyde-3-phosphate, disrupts cellular glycolysis, which in turn leads to eventual cell death, mainly chondrocyte cell death with cartilage degeneration and subsequent subchondral bone alterations^[17]^. Administration of Lpps began in the later stages of the disease (after days 10-14) characterized by progressive cartilage degradation and remodelling of subchondral bone, replacing the first inflammatory episodes. Labelled Lpps were not shown to easily reach the spleen nor the bone marrow close to the subchondral bone in OA rats. At the chronic dosing, it is therefore unlikely that Lpps prevented *in situ* the progression of cartilage degeneration. Moreover, a preliminary investigation of cytokine expression (IL-1, IL-6, and IL-10) in bone marrow APC+ from OA and healthy Lpps-treated rats displayed no significant difference, which further supported the assumption that the subchondral environment was not targeted by Lpps (data not shown).

Actually, Lpps were shown to repress bacteria related to OA, i.e., *P. goldsteinii* and *Akkermansia* within the digest tract. PCA also pointed to *P. goldsteinii* as a contributor to the disease. The involvement of both bacteria in OA progression is unexpected since both bacteria are considered beneficial, even though *P. goldsteinii* can be isolated from infectious samples and *Akkermansia* is enriched in OA obese patients as compared to overweight healthy volunteers^[18-21]^. OA being related to bacterial detection in synovial fluid and tissue, we speculated about a possible enhanced translocation^[22]^. In our study, prevalence of *P. goldsteinii* in Peyer’s patches was similar in both untreated and Lpps-protected rats. Moreover, plasma LPS measurement gave similar values in both groups. But it does not disqualify a possible invasion of the damaged knee through the lymphatic route, *P. goldsteinii* being able to cross the colon to mesenteric nodes and fat in gnotobiotic mice, with possible dissemination through the lymph route^[23]^.

Alternatively, OA progression can be related to both *P. goldsteinii* and *Akkermansia* localized in the lower part of the digestive tract through their common end-products. *P. goldsteinii* mainly produces acetic and succinic acids and minor amounts of isovaleric acid, propionic acid, and formic acid^[24]^. *Akkermansia* is also producing acetate and, to a higher extent, propionate^[25]^. Therefore, decreased populations of *P. goldsteinii and Akkermansia* induced by Lpps intake are likely to result in decreased acetate formation in the lower part of the gut. A salient property of acetate is its ability to effectively promote effector T cells during an active immune response but not in a steady state^[26]^. OA is characterized as a low-grade inflammation. It is thus expected that acetate will promote effector T cells, consequently aggravating cartilage degradation.

Additionally, PCA uncovered a possible involvement of *B.thetaiotaomicron* and *E. plexicaudatum*, as contributing factors to OA prevention. Although there is no significant increase in their population following Lpps intake, the fact that they are connected with the healthy status evokes the possible role of tryptophan catabolism and butyrate formation. L-tryptophan (L-Trp) is associated with the microenvironment of chronic inflammation in OA joints. Tryptophan is a nutritionally essential amino acid that cannot be synthesized *in vivo* and must be provided through dietary sources. In an OA rat model, serum Trp concentration was significantly higher than in the control counterparts^[27]^. Depletion within the gut by increased bacterial catabolism could alleviate cartilage degradation. *B.thetaiotaomicron* breaks down tryptophan into indole, indole-3-acid-acetic (IAA), indole3-lactic acid (ILA), 3-methylindole (skatole)^[28]^. Lpps intake promoted the expansion of *L. murinus*, *L.reuteri* and *L. johnsonii*, all of them producing the bioactive L-Trp metabolite, Indole-3-aldehyde (IAld)^[29]^. Besides, *L.reuteri* produces IAA as L-Trp metabolite. On one hand, the increased catabolism of L-tryptophan is expected to decrease its passage to the bloodstream; on the other hand, L-Trp-derived indole and indole acid derivatives show selectivity to binding and activating the Aryl Hydrocarbon Receptor (AhR). Agonism of the AhR in intestinal epithelial cells can directly enhance barrier function through strengthening of junctional complexes (adherens and tight junctions), expansion of goblet cells and mucus production, increased stem cell turnover, and the promotion of epithelial cell regeneration^[28,29]^. Butyrate acts as a histone deacetylase HDAC inhibitor, leading to increased recruitment of AhR to the target gene promoter in the presence of tryptophan-derived AhR agonists^[30]^. In addition, butyrate was already shown to reduce arthritis severity via the stimulation of AhR in IL-10-producing regulatory B cells^[31]^. Similarly, one might assume that *E. plexicaudatum*, by providing butyrate, helps L-Trp metabolites produced by *B.thetaoiotaomicron* to induce tolerance. Once the homeostasis is broken down by MIA injection, the intestinal barrier is likely more permeable to the passage of luminal antigens and bacteria. We speculate that Lpps, by promoting lactobacilli, provide a supplement in L-Trp metabolites indirectly, likely accentuating the intestinal barrier protection through the AhR pathway. Demonstration in a proteoglycan-induced ankylosis spondylitis mouse model that the AhR pathway is activated by IAA treatment and that the intestinal barrier function is improved further supports our hypothesis^[32]^.

At last, the increase in *M. schaedlerii* following Lpps intake likely also reflected the possible IAA increase related to *L. reuteri* expansion. Administration of IAA led not only to an activation of the AhR pathway but also to an increase in *M. schaedlerii*^[32]^. In turn, *M.shaedlerii* could elicit T-dependent IgA^[33]^. Most commensal bacteria elicit T-independent IgA responses, except *M. schaedlerii*, suggesting in the latter case uptake of the intestinal bacteria by APC^[33]^. Expansion of *M. schaedlerii* could therefore result in immune cell elicitation.

Overall, Lpps primarily protected rats from OA progression through modulation of the microbiota, on one hand, reducing bacteria with a common acetate end-product, and on the other hand, promoting bacteria producing L-Trp metabolites, with a possible reinforcement of the AhR pathway.

At any rate, the promising results demand further investigation focusing on metabolomic analysis. Still, even though the model presents some limitations, our study substantiated the gut-joint axis in the context of OA, opening new avenues for manipulating the microbiome by using bifidobacterial Lpps.

## DECLARATIONS

### Copyright

© The Author(s) 2023.
