# Supplementary material for: Free lipoproteins from Bifidobacterium longum alleviate osteoarthritis through modulation of the gut microbiome
Source: Microbiome Res Rep. 2023 May 11;2(3):18. doi: 10.20517/mrr.2023.12 (PMC10688786; doi:10.20517/mrr.2023.12)
Supplement: Supplementary file 1 [file mrr-2-3-18-SupplementaryMaterials.pdf]

Target species	Sequence 5'-3'	Product size	Annealing	References
All enterococcus	FW: TACTGACAAACCATTCATGATG<br>RV: AACTTCGTCACCAACGCGAAC	112 bp	62°C	Clin et al., 1999
Akkermansia sp.	FW: CAGCACGTGAAGGTGGGGAC<br>RV: CCTTGCGGTTGGCTTCAGAT	329 bp	59°C	Collado et al., 2007
Barnesiella sp.	FW: ACCTTACCCGGGCTCAAAC<br>RV: CTGATGCGCGATTACTAGCGAA	383 bp	58°C	
B.dorei	FW: CTGCCGGCTACAAATTCTACA<br>RV: AGGTTTGAGAAGATGTCGCTC	184 bp	63°C	Cinek et al., 2017
B. fragilis	FW: GGTAACGGCCCACCAAGCCT<br>RV: TGGCTGGTTCAGGCTAACGC	139 bp	62°C	
B. thetaiotaomicron	FW: AACCTTCCTTGCGGGTACAT<br>RV: CGGTGCCTGCTGTGATAAGTA	207 bp	60°C	
B. vulgatus	FW: TGTGCATAGCCGTAATGGTG<br>RV: GTAGCATCCGGCATCCATC	155 bp	63°C	Cinek et al., 2017
Clostridium (ASF356)	FW: CGGTGACTAATACCGCATACGG<br>RV: CCTTGCCGCCTACTCTCCC	413 bp	56°C	
E. coli	FW: TGGGAAGCGAAAATCCTG<br>RV: CAGTACAGGTAGACTTCTG	220 bp	62°C	Maheux et al., 2009
E. faecalis	FW: GCCACTATTTCTCGGACAGC<br>RV: GTCGTCCCTTTGGCAAATAA	518 bp	62°C	Lui et al., 2005
E. plexicaudatum (ASF492)	FW: CTGCGGAATTCCTTCGGGG<br>RV: CCCATACCACCGGAGTTTTC	167 bp	62°C	
L. intestinalis (ASF360)	FW: CTTCGGTGATGACGCTGG<br>RV: GCAATAGCCATGCAGCTATTGTTG	131 bp	62°C	
L. johnsonii	FW: CTCAGGACGAACGCTGGCGG<br>RV: GCAAGCACTAAAATCATCTAGGCAAGC	74 bp	62°C	
L. murinus (ASF361)	FW: GCAATGATGCGTAGCCGAAC<br>RV: GCACTTTCTTCTCTAACAACAGGG	182 bp	63°C	
L. reuteri	FW: ACCGAGAACACCGCGTTATTT<br>RV:	94 bp	61°C	

	CATAACTTAACCTAAACAATCAA AGATTGTCT			
M. schaedleri (ASF457)	FW: CCGAAAGGTGAGCTAATGCCGG RV: GGGACGCGAGTCCATCTTTC	95 bp	57°C	
P. goldsteinii (ASF519)	FW: CACAGTAAGCGGCACAGCG RV: CCGCTCACACGGTAGCTG	428 bp	60°C	Sarma-Rupavtarm et al., 2004
Pseudoflavinofactor sp. (ASF500)	FW: GTCGCATGGCACTGGACATC RV: CCTCAGGTACCGTCACTTGCTTC	285 bp	58°C	

We designed primers except for the few obtained in the literature. No bifidobacteria were recovered using the various primers from our previous works

Table 1: Primers used to quantify ASF bacteria and selected bacteria with a potential role in autoimmune and inflammatory diseases.

**Treatment**	water	Lpps	*p*
**Osteoarthritis**	+	-	
**Rats (n)**	10	8	
**Distal Ileum**			
Ligilactobacillus murinus	5.37 ± 1.5*	7.52 ± 0.6	0.048
Limosilactobacillus reuteri	4.87 ± 0.9	5.96 ± 0.5	0.033
**Caecum**			
Lactobacillus johnsonii	6.19 ± 1.2	7.97± 0.5	0.02
Parabacteroides goldsteinii	8.42 ± 0.6	7.37 ± 0.5	0.021
**Colon**			
L. johnsonii	5.85± 1.5	7.90 ± 0.2	0.008
L. reuteri	5.39 ± 1.5	7.13 ± 0.3	0.023

Mucispirillum schaedleri	5.88 ± 0.7	6.87± 0.4	0.046
Akkermansia sp.	7.41 ± 0.7	6.17 ± 0.5	0.01

*cfu/g intestin

Table 2: Significant variation to intestinal bacteria counts following Lpps intake in MIA-induced OA rats.
